# Bathing in Terminal Care of Cancer Patients and Its Relation to Perceptions of a “Good Death”: A Nationwide Bereavement Survey in Japan

**DOI:** 10.1089/pmr.2021.0075

**Published:** 2022-04-12

**Authors:** Eriko Hayashi, Maho Aoyama, Kento Masukawa, Mitsunori Miyashita, Tatsuya Morita, Yoshiyuki Kizawa, Satoru Tsuneto, Yasuo Shima

**Affiliations:** ^1^ Nursing Course, School of Medicine, Yokohama City University, Yokohama, Kanagawa, Japan.; ^2^ Department of Palliative Nursing, Health Sciences, Tohoku University Graduate School of Medicine, Sendai, Miyagi, Japan.; ^3^ Palliative and Supportive Care Division, Seirei Mikatahara Hospital, Hamamatsu, Shizuoka, Japan.; ^4^ Department of Palliative Medicine, University of Tsukuba Hospital, Ibaraki, Japan.; ^5^ Department of Human Health Sciences, Kyoto University Graduate School of Medicine, Kyoto, Hyogo, Japan.; ^6^ Department of Palliative Medicine, Tsukuba Medical Center Hospital, Tsukuba, Ibaragi, Japan.

**Keywords:** bathing, bathing in a tub, bereaved family, good death, palliative care ward, terminal cancer patient

## Abstract

**Background:**

Bathing in a tub is integral to Japanese culture. It improves palliative care patients' symptoms and may improve quality of life.

**Objectives:**

This study aimed to determine the prevalence and impressions of bathing for terminally ill cancer patients and its relations to the evaluations of perceived end-of-life care and achievement of a good death.

**Design:**

This was a cross-sectional, anonymous, self-report questionnaire survey.

**Setting/Subjects:**

The questionnaire for this study was sent to bereaved family members who had lost loved ones in 14 general hospitals and 187 palliative care wards in Japan.

**Measurements:**

The bereaved family members of the patients who had actually bathed were asked about their impression of bathing. The short version of the Good Death Inventory (GDI) and the Care Evaluation Scale were used to evaluate “achievement of a good death.” In total, 1819 surveys were sent between July and September 2018 to bereaved family members of patients who had died between February 2014 and January 2018 in 14 general hospitals and 187 palliative care wards in Japan. Overall 885 questionnaires (valid response rate 48%) returned by bereaved family members were analyzed.

**Results:**

Overall, 85% of bereaved family members of patients who bathed evaluated the experience positively, 86% reported that the patient's face seemed to become calm after the bath, and 28% of bereaved family members whose loved one had not bathed reported regretting it. The total GDI score for the bereaved family's desired death was 82.7 ± 13.0 for the bathing group and 75.4 ± 15.7 for the no bathing group, a significant difference (effect size = 0.52, *p* < 0.01).

**Conclusions:**

Bathing before death was evaluated positively and was associated with the achievement of a good death.

## Introduction

Bathing in a tub (hereinafter “bathing”) is an important custom and an integral behavior of Japanese culture, as well as of other cultures. The purposes of bathing and showering are different for Japanese people. In addition, a previous survey reported that >90% of Japanese people are not satisfied with only taking a shower.^1,2^ The main purposes of bathing are relaxation, relieving fatigue, and warming the body, whereas showering is mainly for washing and cleaning the body.^1,3^ This custom is also considered essential even at the end of life. Skaczkowski et al.^4^ reported that spa baths improved palliative care patients' self-reported pain, anxiety, and well-being, which may improve quality of life. Moreover, spa baths may be simple and effective care within the normal course of nursing duties without the need for additional training.^4,5^

End-of-life cancer patients experience a decrease in activities of daily living (ADL) and in cognitive function from two weeks before their death, and multiple symptoms such as malaise and pain appear.^5^ Therefore, there is concern that bathing could be invasive care for them. There have been some international studies, some of which have reported the safety and efficacy of bathing for terminally ill patients.^3,4,6–14^ Fujimoto reported that bathing was a safe and comfortable care practice for terminally ill patients, because it did not cause significant fluctuations in circulatory dynamics, and it reduced anxiety.^11^ In the author's preliminary study, a comparison of before and after bathing for terminal cancer patients showed that it was safe and reduced fatigue.^15^ According to another interview survey in Japan, end-of-life cancer patients reported that bathing was a very peaceful experience and was effective to release them from their symptoms.^16^ Therefore, bathing could be considered a meaningful daily care practice provided by nurses for terminally ill patients. However, the author's preliminary study has not been confirmed by multicenter studies or by studies involving bereaved families.

Although it is unclear how frequently bathing is provided for terminally ill patients, it is also important to define what bathing for these patients entails and to determine its clinical necessity and utility. Furthermore, given the importance of this custom in Japanese culture, providing bathing care might improve both patient and family satisfaction with the patient's quality of life and end-of-life care.

## Methods

This study was part of the fourth Japan Hospice and Palliative Care Evaluation study,^17^ one of the projects of the Japan Hospice and Palliative Care Research Foundation (J-HOPE). The details of the design and procedure of the study were described in its protocol paper.^17^ In brief, a cross-sectional, anonymous, self-report questionnaire survey was conducted between July and September 2018. The questionnaire for this study was sent to the bereaved families who lost loved ones in 14 general hospitals and 187 palliative care wards that were members of the Japan Hospice and Palliative Care Association as of July 2017.

To identify potential subjects, participating institutions were asked to list up to 80 bereaved family members of patients who had died before January 2018. The inclusion criteria were as follows: (1) the patient died of cancer; (2) the patient was at least 20 years old (the age at which one is considered an adult in Japan); and (3) the bereaved family member was at least 20 years old. The exclusion criteria were as follows: (1) the patient received palliative care for less than three days; (2) the bereaved family member was unavailable or could not be identified; (3) treatment-associated death or death in an intensive care ward; (4) the potential participant would have suffered serious psychological distress, as determined by the primary physician and a nurse; and (5) the potential participant was incapable of completing the self-report questionnaire because of health issues, such as cognitive impairment or a visual disability.

Questionnaires were sent to the bereaved family members identified by each participating institution. A document explaining the aims and procedures of the J-HOPE4 study was sent with the questionnaire, and the return of a completed questionnaire was considered to indicate consent to participate in the study. Ethical approval for the study was granted by the institutional review boards of Tohoku University (No. 2017-2-36-1) and all participating institutions.

### Mechanical bath in a palliative care ward

**FIG. 1.**
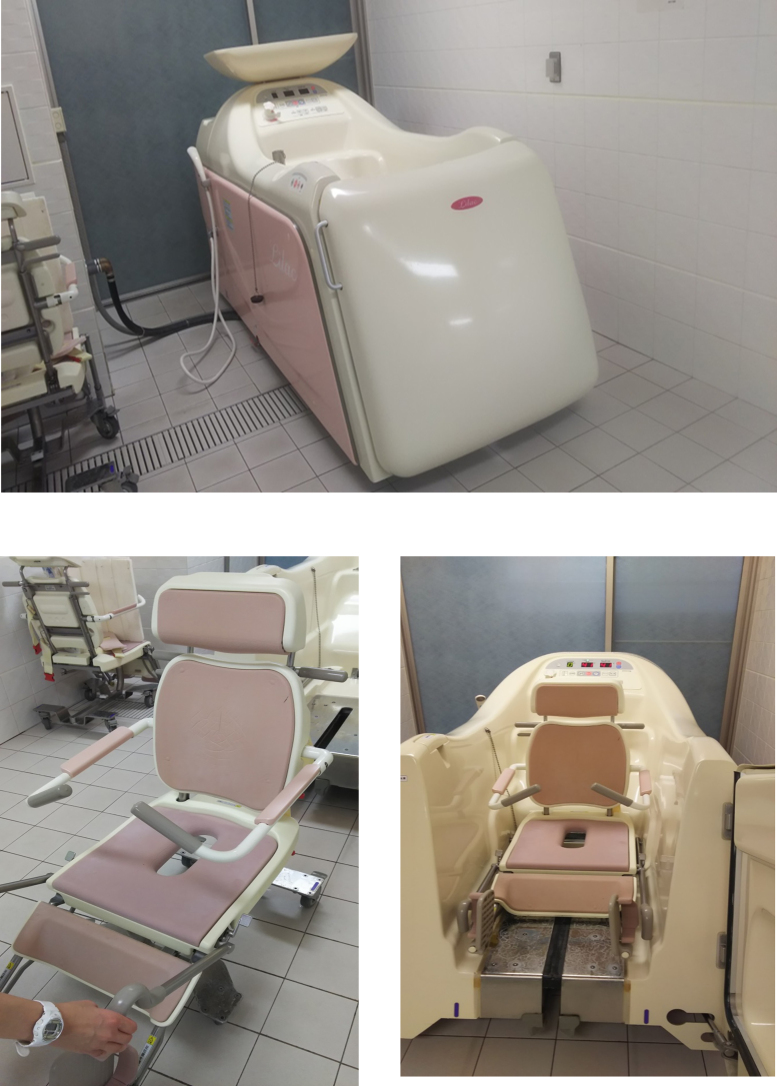
Mechanical bath in a palliative care ward. It is possible for the patient to take a bath in a sitting position or lying down, when activities of daily living, energy, and the physical function of terminally patients are at their weakest. The temperature of the hot water can be kept constant. The photograph shows a position close to bed rest, and the bathtub is filled about two to three minutes after the patient enters the bathtub. There is a bubble function by default. Bathrooms equipped with mechanical baths have ample space around the bathtub for caregivers to facilitate bathing assistance. There is a heater to keep the body warm when taking a bath, and a toilet is also installed.

Most hospital and elderly nursing facilities in Japan have mechanical baths. A mechanical bath is for people who cannot move, and it fills automatically with hot water when the patient enters the bathtub in a reclining wheelchair or bed (Fig. 1).

### Measurements

#### Experience of bathing and impressions of the bereaved families

Whether the patient bathed during the last hospitalization was asked by a single question: “When was the last time the patient took a bath in a tub at the place of death (hospital)?” Responses could be chosen from the following categories: “Within 2 weeks before death,” “One month to 2 weeks before death,” “More than 1 month before death,” “Never during the hospital stay,” and “I don't know.” In addition, the participants were asked about patient's preference and frequency of bathing while healthy. The participants were dichotomized according to whether the patients had bathed in the end-of-life period based on the answer to their last bath in a tub as: “within 2 weeks/2 weeks to 1 month ago” and “more than 1 month ago/not in the hospital.”

#### Impressions of the bereaved families of patients' bathing

The bereaved families of the patients who actually had bathed were asked about their impression of bathing. The questionnaire items included impressions of the patient, impressions of the bereaved family, effects of bathing, effects on relief of pain and psychological symptoms, and changes after bathing. In addition, bereaved family members were asked to respond to the following statements using a 5-point scale ranging from “Strongly disagree” to “Strongly agree”: *as a family, we were surprised the patient could bathe in this condition; as a family, we were happy to feel like we were treated with care; the patient enjoyed and appreciated “bathing”; I regret the patient “bathed”; and the “bath” hastened his death and made his condition worse.*

For the bereaved families of the patients who had not bathed in their last 30 days, the following four items were asked with a 5-point scale ranging from “Strongly disagree” to “Strongly agree”: *patients could not bathe due to their poor condition; the patient's body was bothered by the dirt; they wished the patient could have bathed before dying; and I still regret the patient was not able to “bathe.”*

#### Achievement of a good death

The short version of the Good Death Inventory (GDI) was used to measure patients' “achievement of a good death” from the perspective of bereaved family members. The short version of the GDI consists of 18 items, with a higher total score indicating the achievement of a good death. The validity and reliability of the scale have been confirmed.^18,19^

#### Care Evaluation Scale version 2

The revised short version of the Care Evaluation Scale (CES) was used in this study. The CES was developed to measure end-of-life care from the perspective of bereaved families, with a focus on the structure and process of care. The short version of the CES consists of 10 representative items scored using a 6-point Likert scale. Higher scores indicate better care. The validity and reliability of the scale have been confirmed.^19,20^

#### Overall care satisfaction

Participants were asked about their overall satisfaction with the care the patient had received at the place of death. The question asked was, “Overall, were you satisfied with the medical care the patient received?” Participants were asked to respond using a 6-point Likert scale (1: absolutely dissatisfied, 2: dissatisfied, 3: somewhat dissatisfied, 4: somewhat satisfied, 5: satisfied, and 6: absolutely satisfied).^19^

### Analysis

First, a descriptive analysis of the bereaved family member's demographic background characteristics, experiences and impressions of bathing among the bereaved families, evaluation of perceived care at the place of death (CES2), and achievement of a good death was conducted. Subsequently, bivariate analyses using the chi-squared test, Fisher's exact test, and the Wilcoxon rank-sum test were conducted to clarify the relationships between bathing and evaluations of perceived care at the place of death and achievement of a good death. Effect size (ES) was calculated based on ϕ and V defined by Cramer et al.^21^: small ES, 0.10; medium ES 0.30; and large ES 0.50. A *p*-value <0.05 was considered significant, and all tests were two-tailed. SPSS 26.0 Japanese version for Windows (SPSS Inc., Chicago, IL) was used for statistical analysis.

## Results

**FIG. 2.**
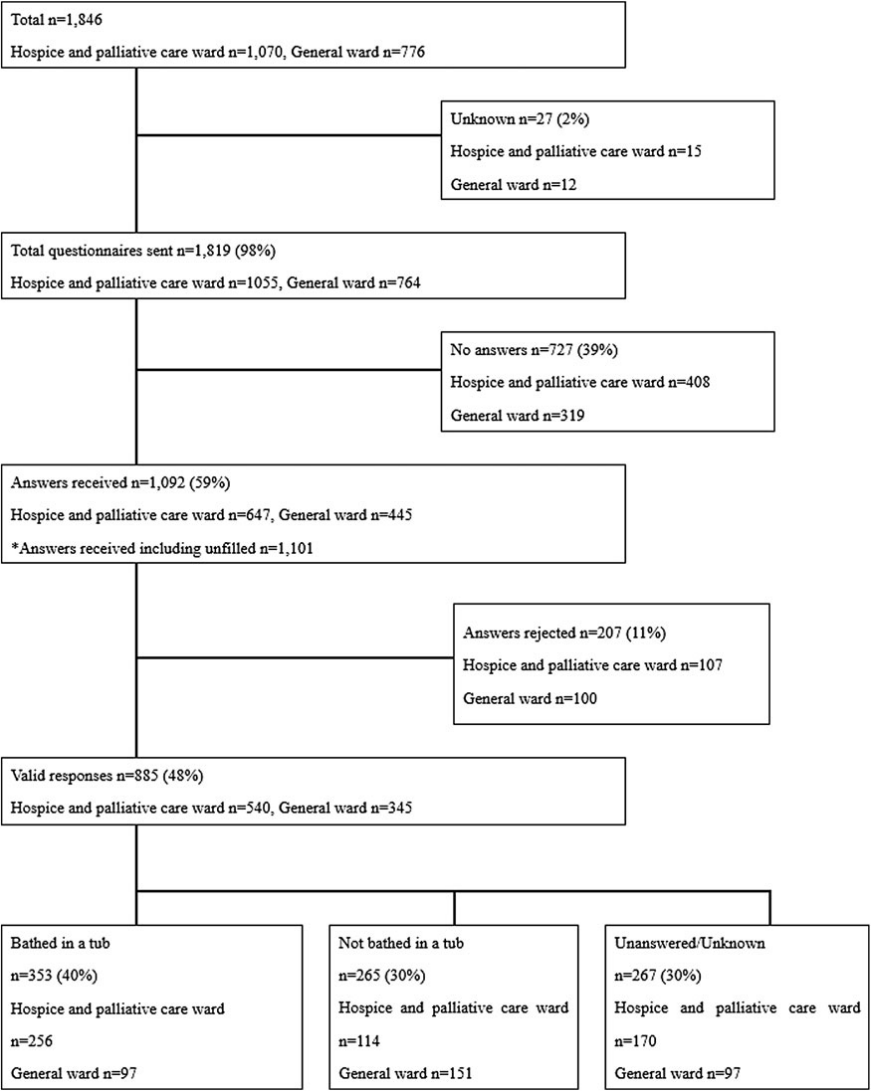
Enrollment of study participants.

Overall 885 questionnaires (valid response rate 48%) from the bereaved families were analyzed (Fig. 2).

The participants' characteristics and whether the patient bathed are given in Table 1. Overall, 56% (*n* = 344) of the patients were men, and the average age (±standard deviation) was 74.7 ± 11.5 years. Regarding the relationship to the patient, 51% (*n* = 313) of participants were spouses, and 35% (*n* = 214) were the patient's child.

**Table 1.** Attributes of Patients' and Bereaved Families With and Without Patients Bathing

	Overall		Bathing group		No bathing group				
	*n* = 618	%	*n* = 353	%	*n* = 265	%	ES	*p*	
Patients' characteristics									
Sex									
Male	344	56	200	57	144	54	0.00	0.57	a
Female	274	44	153	43	121	46			
Age (years)									
Mean ± SD	74.7 ± 11.5		74.3 ± 12.2		73.5 ± 11.9		0.07	0.73	b
Cancer site									
Lungs	104	17	55	16	49	19	0.04	0.04	a
Liver, pancreas, gallbladder, bile duct	133	22	92	26	41	16			
Esophagus and stomach	88	14	49	14	39	15			
Colon and rectum	83	13	48	14	35	13			
Kidney, bladder, prostate	42	7	24	7	18	7			
Breast	34	6	17	5	17	6			
Uterus/ovary	37	6	23	7	14	5			
Other	97	16	45	13	52	20			
Time to death									
Mean ± SD	35.6 ± 48.8		40.1 ± 58.9		27.3 ± 29.6		0.26	<0.01	b
Less than one week	86	14	33	9	28	11	0.04	<0.01	a
More than one week and less than two weeks	116	19	54	15	37	15			
More than two weeks and less than one month	176	29	111	31	73	29			
More than one month	240	38	155	43	110	44			
Bereaved families' characteristics									
Sex									
Male	202	32	122	35	80	30	0.01	0.47	a
Female	414	67	230	65	184	70			
Age (years)									
Mean ± SD	62.4 ± 11.7		62.5 ± 11.5		62.2 ± 12.0		0.03	0.73	b
Relatives									
Spouse	313	51	171	49	142	55	0.01	0.26	a
Child	214	35	127	36	87	34			
Other	84	14	53	15	31	12			
The family stayed in the hospital for a week until the patient died							0.05	0.01	a
Daily	434	71	229	65	205	77			
Four to six days	96	16	68	19	28	11			
One to three days	65	11	42	12	23	9			
Did not stay at the hospital	21	3	12	3	9	3			
Preference for “bathing in a tub” of alive patients									
Preference for “bathing in a tub” when your patient was healthy							0.02	0.18	a
I think so	516	85	304	87	212	82			
Neither	49	8	23	7	26	10			
I do not think so	45	7	23	7	22	9			
Frequency of “bathing in a tub” when your patient was healthy							0.07	<0.01	a
Daily	327	53	167	48	160	61			
One to three times a week	241	39	161	46	80	30			
One to three times a month	29	5	21	6	8	3			
Not in/not sure	17	3	2	1	15	6			

^a^ Fisher's exact test.

^b^ Continuous variables were assessed using the Wilcoxon rank-sum test, with *p* < 0.05 considered significant.

ES, effect size; SD, standard deviation.

The percentage of participants who answered that the patients preferred bathing when they were healthy was 85% (*n* = 516). In addition, regarding the frequency of bathing, 53% (*n* = 327) reported “every day,” followed by 39% (*n* = 241) responding “once to three times a week.” There was no significant relationship between the preference and frequency of bathing when healthy and whether they actually had bathed in their last 30 days. There was a significant relationship between bathing and length of hospitalization in the place of death (bathing group vs. no bathing group: 40.1 ± 58.9 days vs. 27.3 ± 29.2 days, ES = 0.26; *p* < 0.01). A significant relationship was observed between bathing and the frequency of daily bathing when the patient was healthy (bathing group vs. no bathing group: 48% vs. 61%, ES = 0.07; *p* < 0.01).

Table 2 provides the distribution of the impressions of bathing of bereaved family members. Of the family members who reported that the patient bathed, 89% (*n* = 282) reported that they were glad to see the patient treated well by bathing, 84% (*n* = 270) reported that the patients enjoyed bathing, and 86% (*n* = 276) reported that the patient's face seemed to become calm after the bath. In addition, 73% (*n* = 230) agreed with the statement, “As a family, we were surprised that the patient could bathe in such a severe condition.” With regard to regret of the bereaved family of the patients who bathed, 4% (*n* = 13), 3% (*n* = 7), and 2% (*n* = 6), respectively, agreed with the following statements: “There was a negative change in the patient after bathing”; “I regret that the patient took a bath”; and “Bathing worsened the patient's condition.” A significant difference was observed between the palliative care ward and the general ward for “After a bath, the patient's physical pain eased” (ES = 0.00, *p* = 0.02).

**Table 2.** Impressions of Bereaved Family Members Whose Patients Bathed in a Tub

	Hospice and palliative care ward		General ward		ES	*p*
	*n* = 256	%	*n* = 97	%		
Overall care						
As a family, we were surprised that the patient could bathe in this condition	177	77	53	62	0.95	0.96
Family members were glad to see the patient being treated well by bathing	216	93	66	77	0.62	0.48
Good experience (positive)						
The patient enjoyed bathing	197	85	73	84	0.19	0.19
The patient's face seemed to become calm after bathing	205	86	71	83	0.18	0.54
After a bath, the patient's physical pain eased	149	64	50	57	0.00	0.02
After a bath, the patient's mind calmed down	184	79	68	78	0.18	0.15
There was a positive change in the patient after bathing	141	62	54	63	0.04	0.11
Bad experiences (negative)						
There was a negative change in the patient after bathing	10	4	3	4	0.47	0.56
I regret that the patient took a bath	5	2	2	2	0.90	0.90
Bathing had worsened the condition of the patient	4	2	2	2	0.74	0.74

Percentage of strongly agree/somewhat agree with 5 grades of answers. Fisher's exact test, with *p* < 0.05 considered significant.

Table 3 provides the distribution of the impressions of the bereaved family members of patients who had not bathed. Overall, 84% (*n* = 206) reported that the patient was too ill for bathing, 61% (*n* = 144) reported that they wished the patient could have bathed before dying, and 28% (*n* = 67) reported that they regretted that the patient could not bathe before death. No significant difference was observed for the impression of the patients' bathing between the hospice palliative care ward and the general ward in the group without bathing experience.

**Table 3.** Impressions of Bereaved Family Members Whose Patients Had Not Bathed in a Tub

	Hospice and palliative care ward		General ward		ES	*p*
	*n* = 114	%	*n* = 151	%		
The patient was too ill for bathing	90	85	116	83	0.74	0.82
They felt the patient was not clean enough	31	31	45	33	0.18	0.18
They wished the patient could have bathed before dying	63	62	81	59	0.24	0.25
They regret that the patient could not bathe before dying	24	24	43	31	0.65	0.77

Percentage of strongly agree/somewhat agree in 5 grades of answers. Fisher's exact test, with *p* < 0.05 considered significant.

The relationships between bathing and achievement of a good death and overall care satisfaction are given in Table 4. The total GDI score for the bereaved family's desired death was 82.7 ± 13.0 for the bathing group and 75.4 ± 15.7 for the no bathing group, a significant difference (ES = 0.52, *p* < 0.01). There was a significant difference in the CES total score between the bereaved families of patients who had (48.7 ± 9.3) and had not (45.5 ± 9.5) bathed in their end-of-life period (ES = 0.34, *p* < 0.01). For overall care satisfaction, there was a significant difference between the bereaved family members of patients who had and had not bathed, with satisfaction reported by 94% (5.1 ± 1.0) and 84% (4.6 ± 1.2) (ES = 0.40, *p* < 0.01), respectively.

**Table 4.** Factors Associated with Care and Satisfaction of Bereaved Families with the Patient Bathing

	Bathing group, n = 353		No bathing group, n = 265		ES	*p*
	Hospice and palliative care ward	General ward	Hospice and palliative care ward	General ward		
	*n* = 256	*n* = 97	*n* = 114	*n* = 151		
GDI total scores (18 items)	82.7 ± 13.0		75.4 ± 15.7		0.52	<0.01
	83.5 ± 13.0		80.3 ± 13.2		0.24	0.01
		80.6 ± 12.7		71.6 ± 16.4	0.52	<0.01
CES total scores	48.7 ± 9.3		45.5 ± 9.5		0.34	<0.01
	49.5 ± 8.8		47.9 ± 8.6		0.20	0.03
		47.8 ± 8.2		43.7 ± 9.8	0.29	<0.01
Overall satisfaction	5.1 ± 1.0		4.6 ± 1.2		0.40	<0.01
	5.2 ± 0.9		5.1 ± 0.9		0.12	0.21
		4.9 ± 1.0		4.3 ± 1.3	0.21	0.01

Continuous variables were assessed using the Wilcoxon rank-sum test, with *p* < 0.05 considered significant.

CES, Care Evaluation Scale; GDI, Good Death Inventory.

## Discussion

The main findings of this study were as follows: (1) the percentage of terminally ill cancer patients who bathed during the four weeks before death was 47% in hospice/palliative care wards and 28% in general wards; (2) ∼85% of bereaved families of patients who bathed evaluated the experience positively, with 86% reporting that the patient's face seemed to become calm after the bath; (3) of the bereaved families of patients who had not bathed, 28% reported that they regretted that the patient had not bathed; and (4) there was a significant association between bathing and a positive evaluation of end-of-life care and the achievement of a good death.

First, of the participants in this study, 85% (*n* = 516) reported that the deceased patients preferred bathing when they were healthy. This is consistent with the previous reports that Japanese people have a high need for bathing.^1,3,6,9–11,15,16^ However, it is often difficult for terminally ill patients, especially in their last month, to bathe independently because of their severe symptoms and decreased ADL, because they require the assistance of two or more nurses to take a bath.^4,11,14,16,22,23^ Although there is a high need for bathing even among terminally ill cancer patients, in this study, only 40% (*n* = 353) of the patients could actually bathe. This gap might be owing to terminal cancer patients being in poor condition, having a short hospital stay, and limitations of the facilities, such as a limited number of nursing staff. Overall, 85% of people preferred bathtub bathing, but, in their last month, their condition was poor or the number of medical staff was limited, and bathing was difficult. However, the results suggest that palliative care wards in Japan are making great efforts to provide machine baths, because it is usually difficult to provide bathing care in general wards owing to a lack of personnel and the environment.

Second, we suggest that bathing is a care practice that allows patients to feel themselves valued as human beings, with the family having the same perspective. In this study, 73% of the bereaved families whose patients bathed reported that they were surprised that the patients could bathe although they were in a severe condition, and 89% reported that they were glad that the patient was treated well, and bathing was a thoughtful care practice. These results suggest that bathing was an important experience for terminally ill cancer patients and their families who could feel that the patients were valued as persons and that their dignity was respected. In addition, only a few bereaved families reported that they felt regret or other negative impressions related to the experience of the patient bathing. They had an impression that it worsened the patient's physical condition and hastened death.

Moreover, of the bereaved families of patients who had not bathed in the place of death, 61% reported that they had wished that the patient had bathed, and 28% reported that they regretted that the patient could not bathe. The bereaved families of patients who had not bathed were more likely to recall that the patients bathed daily when healthy. They were most likely recalling and describing bathing when the patients were healthy. The patients used to bathe daily, but were not able to do so during the month of their death.

Therefore, it is considered that the bereaved families in the no bathing group wished that the patients could have experienced it more. In clinical settings, bathing tends to be avoided for terminal cancer patients because of the severity of the illness and the physical burden. However, the present results show how impressed the families were when terminal cancer patients received bathing care even when the patients' condition was poor. Moreover, considering dignity, maintaining a clean body contributes to the patient's comfort and the family's feeling that the patient is well cared for.^24–26^ Therefore, we suggest that offering a bath in a tub may be a meaningful care practice not only for terminal cancer patients, but also for their families.

Third, another key finding of the study was the relationship between bathing and the quality of palliative care. Bereaved families whose patients had bathed reported higher GDI and CES scores than those who had not. In addition, the bereaved families whose patients had bathed reported less regrets regarding whether the patients did what they wanted to do to the end. From the perspective of achievement of a good death, bereaved families of those patients who took a bath tended to report that the patients were able to spend time where they wanted; more specifically, they had some enjoyment until the end in a home-like environment.^27^

The present findings suggest that bathing is simple but effective nursing care for terminal cancer patients that can be included in daily duties by adding the family's perspective.^4^ The bathing itself may not have been directly related to the GDI or CES scores, but other factors, such as enthusiastic care in providing a bath, may have resulted in a good evaluation of the overall care. In addition, many of the people who did not take a bath seemed to have suddenly deteriorated, and it is possible that their hospitalization period was short, and therefore the quality of care was evaluated as low.

### Limitations

This study has several limitations. First, the response rate was 48%, and ∼30% of the respondents were excluded from the analysis because they did not know whether the patient had bathed in the last month of life. Second, because this was a cross-sectional study, causal inferences between variables cannot be made. Future studies with longitudinal designs are needed to clarify the causal relationships between variables. Third, the quality of end-of-life care and the achievement of a good death were assessed by the bereaved family retrospectively, which may have led to recall bias. Fourth, this study involved subjects from 150 hospitals and is highly representative, but there was bias owing to subjects coming from 20 general facilities.

## Conclusion

Approximately over one-third of cancer-bereaved families whose patients had bathed felt it was “thoughtful care,” a “good experience,” or a “positive experience.” There was a significant association between bathing and a positive evaluation of the quality of palliative care and the achievement of a good death.

## Availability of Data and Material (Data Transparency)

The datasets and/or analyses of this study are available from the corresponding author on reasonable request.

## Abbreviations Used

ADL: activities of daily living
CES: Care Evaluation Scale
ES: effect size
GDI: Good Death Inventory
J-HOPE: Japan Hospice and Palliative Care Research Foundation
SD: standard deviation
